# Supplementary material for: LPP is a Src substrate required for invadopodia formation and efficient breast cancer lung metastasis
Source: Nat Commun. 2017 Apr 24;8:15059. doi: 10.1038/ncomms15059 (PMC5413977; doi:10.1038/ncomms15059)
Supplement: Supplementary Information — Supplementary Figures [file ncomms15059-s1.pdf]

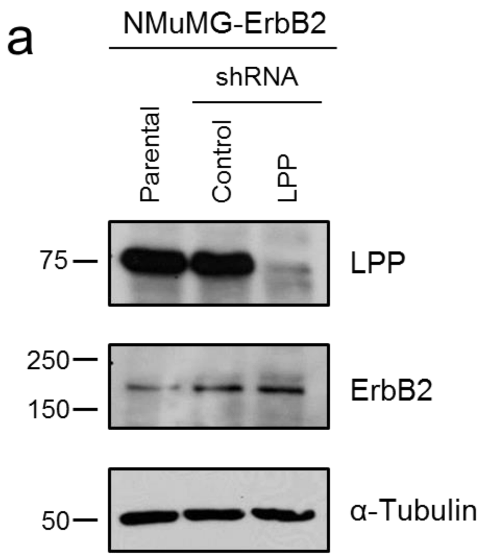

Supplementary Figure 1. Characterization of NMuMG-ErbB2 and NIC breast cancer cells expressing shRNAs targeting LPP. NMuMG-ErbB2 cells (a) and NIC cells (b) were engineered to stably express either a LucA-shRNA (Control) or a shRNA targeting LPP. Immunoblot analyses of LPP and ErbB2 expression are shown. α-Tubulin was used as a loading control.

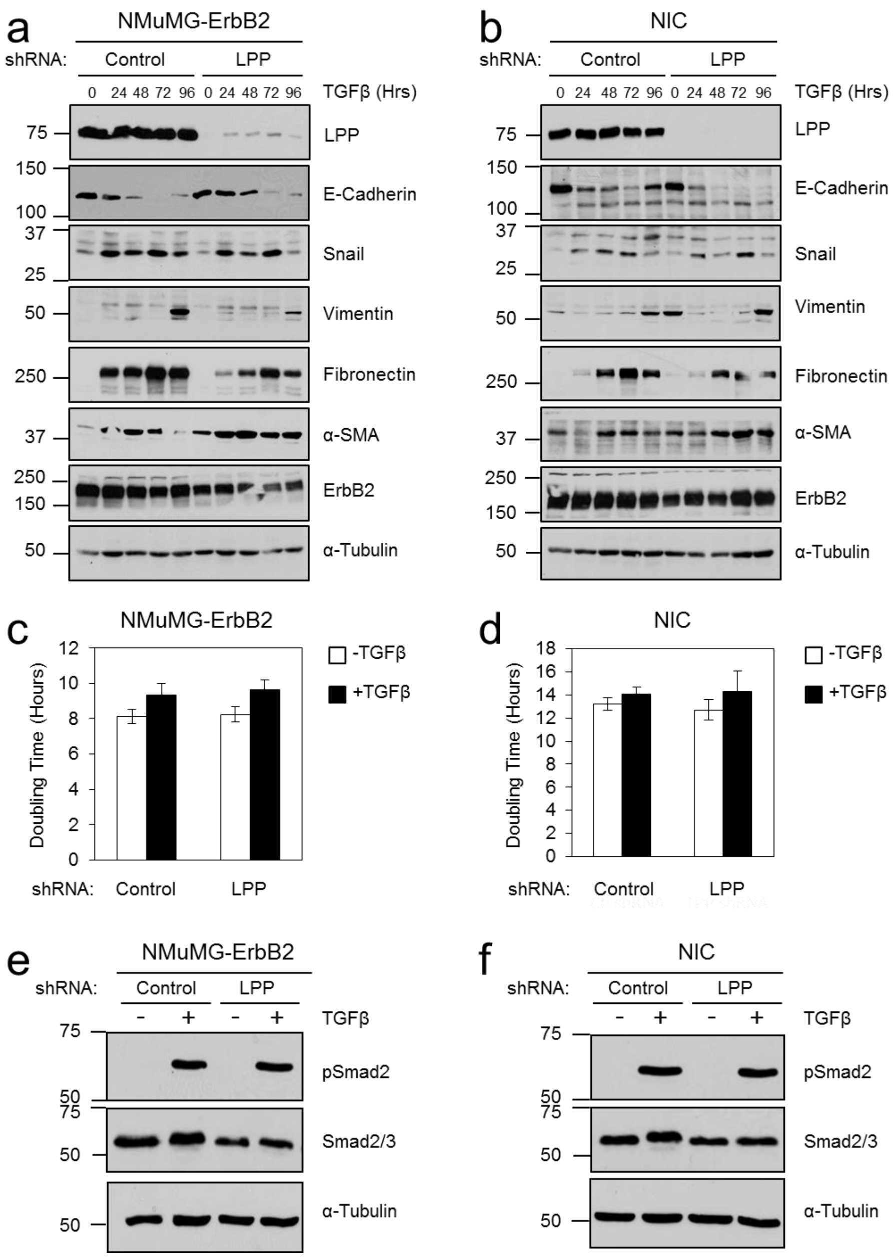

Supplementary Figure 2. Reduced LPP expression does not alter breast cancer cell proliferation or their ability to undergo TGFβ-induced EMT. NMuMG-ErbB2 and NIC breast cancer cells expressing control shRNA (Luc-shRNA) or LPP-targeting shRNA (LPP-shRNA) were treated with or without TGFβ for the indicated times. (a, b) Immunoblot analyses of NMuMG-ErbB2 and NIC breast cancer cell populations were performed to assess expression of LPP, ErbB2, an epithelial marker (E-cadherin) and mesenchymal markers (Snail, Vimentin, Fibronectin and α-SMA) following TGFβ stimulation. TGFβ was refreshed after 48 hours. α-Tubulin was used as a loading control. (c, d) NMuMG-ErbB2 and NIC cell populations were subjected to proliferation assays in the presence or absence of TGFβ. (e, f) NMuMG-ErbB2 and NIC cell populations harboring an LPP knockdown were assessed for Smad2 phosphorylation and total Smad2 levels following 30 minutes of TGFβ-stimulation. α-Tubulin was used as a loading control.

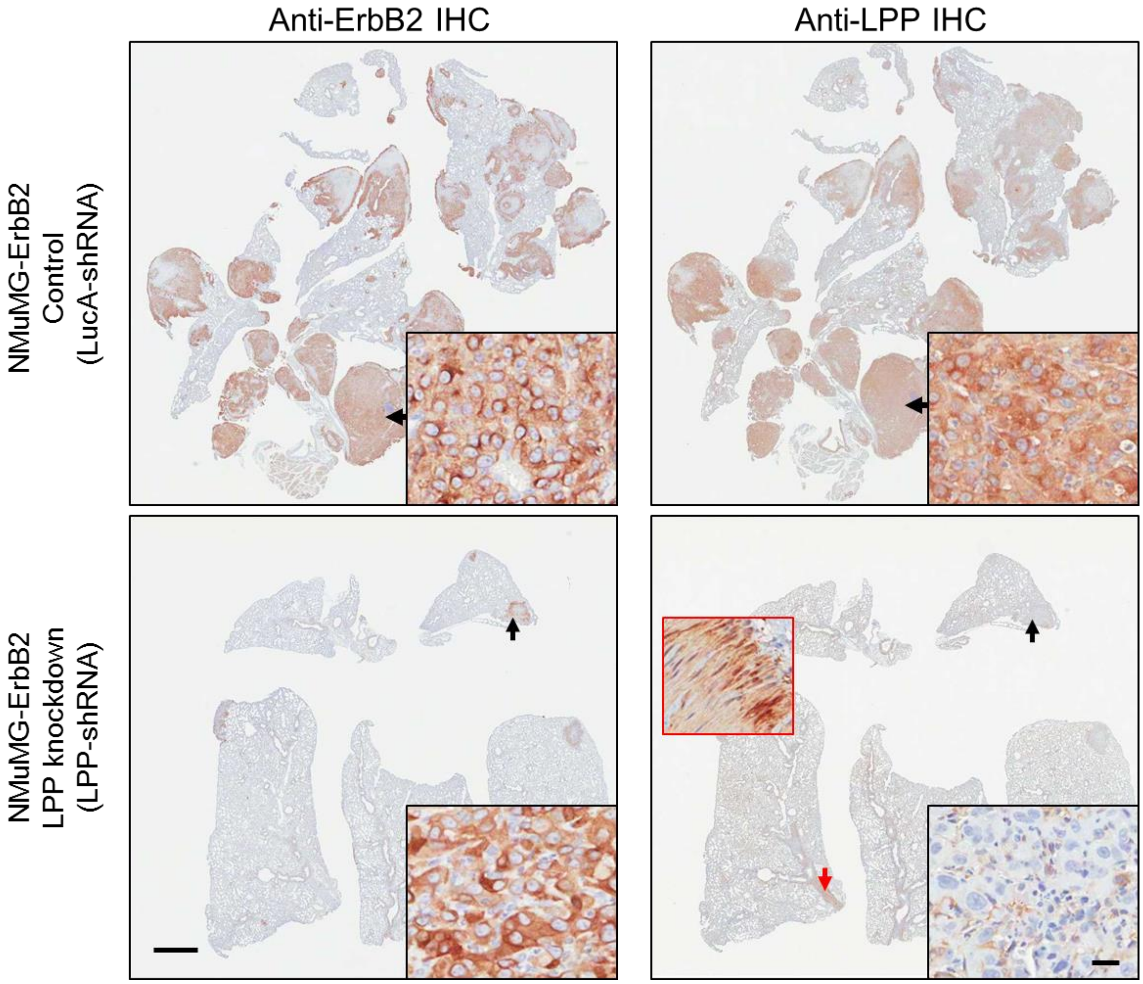

Supplementary Figure 3. Immunohistochemical staining of LPP and ErbB2 in lung metastases. NMuMG-ErbB2 cells harboring stable LPP-targeting (LPP-shRNA) or LucA control shRNAs (LucA-shRNA) were injected into the mammary fat pads of mice. Lungs were collected at necropsy and stained for the expression of ErbB2 and LPP within the lung metastatic lesions. An area containing smooth muscle, taken from the same section, shows strong endogenous LPP staining (red box) and serves as a positive control for the IHC staining. The arrows indicate regions within the low magnification images that are shown in the insets. The scale bar within the low magnification images represents 2 mm and applies to all images. The scale bar within the inset represents 20μm and applies to all inset images.

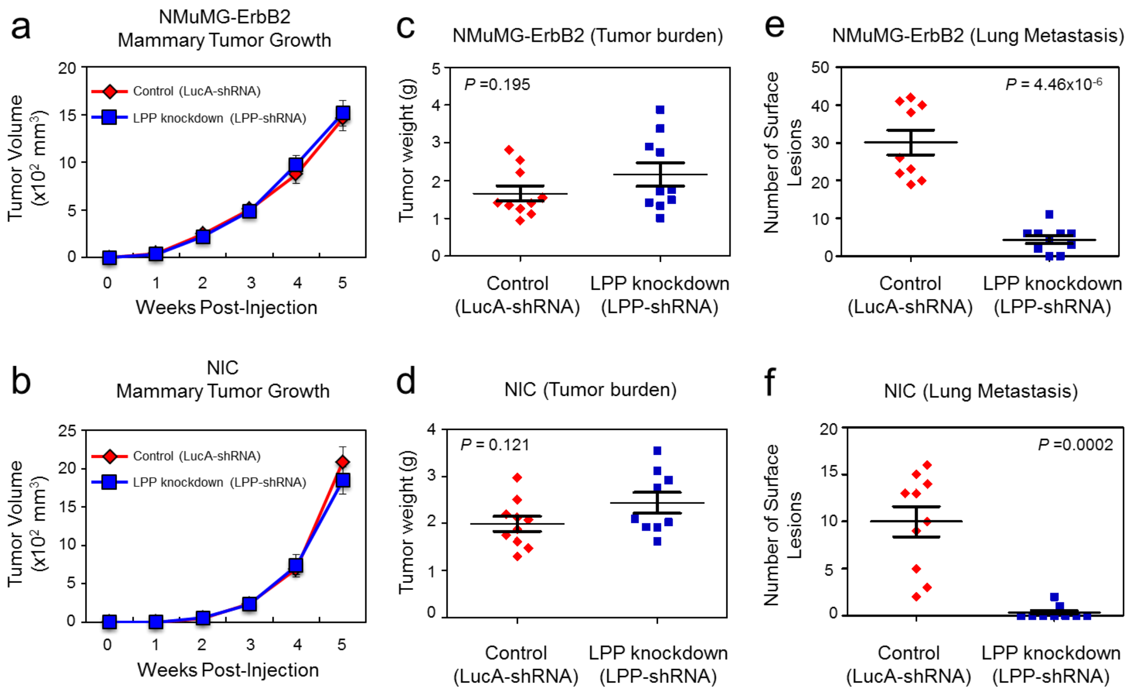

Supplementary Figure 4. Reduced LPP does not affect tumor burden but decreases breast cancer metastasis to the lung. Tumor growth of mice injected with (a) NMuMG-ErbB2 breast cancer cells harboring LucA- or LPP-shRNAs, or (b) NIC breast cancer cells harboring LucA- or LPP-shRNAs was determined by weekly caliper measurement over 5 weeks. At the time of sacrifice, whole blood was collected for CTC analysis and tumors from NMuMG-ErbB2 and NIC animals were weighed to determine the final tumor burden (c and d). Lung tissue was also collected at necropsy and the number of macroscopic lesions on the lung surfaces were quantified from cohorts of mice injected with (e) NMuMG-ErbB2 breast cancer cells harboring LucA- (n=9) or LPP-shRNAs (n=10) or (f) NIC breast cancer cells possessing LucA- (n=10) or LPP-shRNAs (n=10). Error bars represent s.e.m. for all panels.

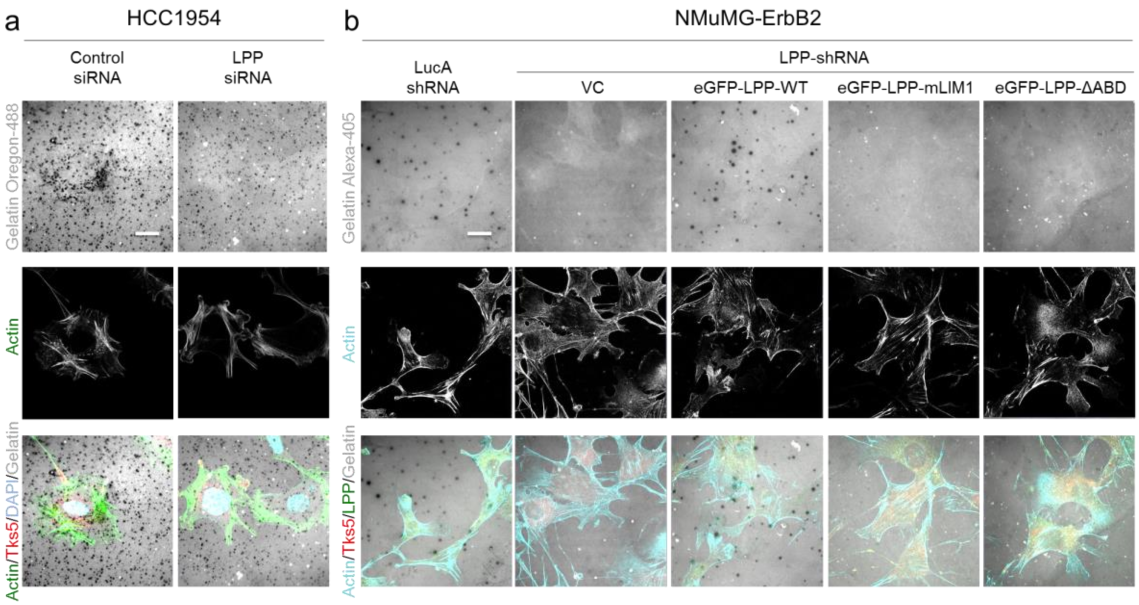

Supplementary Figure 5. LPP expression is required for degradation of ECM by breast cancer cells. (a) NMuMG-ErbB2 cells expressing LucA-shRNA (control) and LPP-shRNA (LPP knockdown), along with rescue constructs (Vector Control: VC, eGFP-LPP-WT, eGFP-LPP-LIM1 or eGFP-LPP-ΔABD), were treated with TGFβ (24 hours) and plated onto Alexa 405 labeled gelatin. After 24 hours, cells were fixed and stained for LPP (in control and VC cells), Tks5 and Actin (phalloidin) to visualize their localization on the gelatin substrate. (b) HCC1954 breast cancer cells were transfected with either scrambled or LPP targeting siRNAs. Cells were treated with TGFβ (24 hours) and subsequently plated onto Oregon 488 labeled gelatin for 24 hours prior to fixation. Coverslips were stained with antibodies against Tks5 and Actin (phalloidin). DAPI was used to visualize the nucleus. Scale bar = 20μm, and applies to all images.

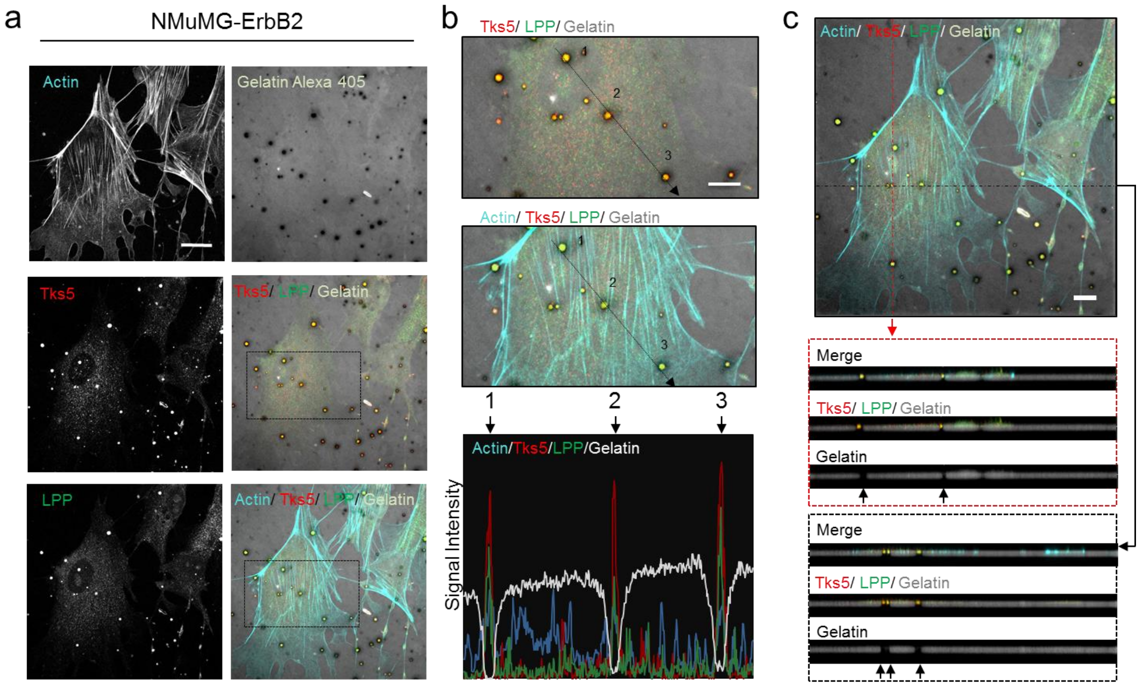

Supplementary Figure 6. LPP co-localizes with Tks5 and Actin within active invadopodia. NMuMG-ErbB2 breast cancer cells were pre-treated with TGFβ (24 hours) prior to plating onto Alexa 405 conjugated gelatin. Cells were fixed after 24 hours and stained with antibodies against LPP, Tks5 and actin (phalloidin). (a) Confocal images were acquired to visualize gelatin degradation, and the localization of Actin, Tks5 and LPP, Scale bar = 20μm and applies to all images. (b) Linescan analysis was performed over areas of degraded gelatin (indicated by black arrow) and the signal intensity of Actin, Tks5, and LPP are shown for three regions of interest. Scale bar = 10μm. (c) Z-stack acquisition was performed over 6.0μm depth at 0.20μm intervals. Orthogonal views (y-z plane: red box; x-z plane: black box) are presented. Black arrows indicate areas of gelatin degradation where LPP, Tks5 and actin are co-localized. Scale bar = 10μm.

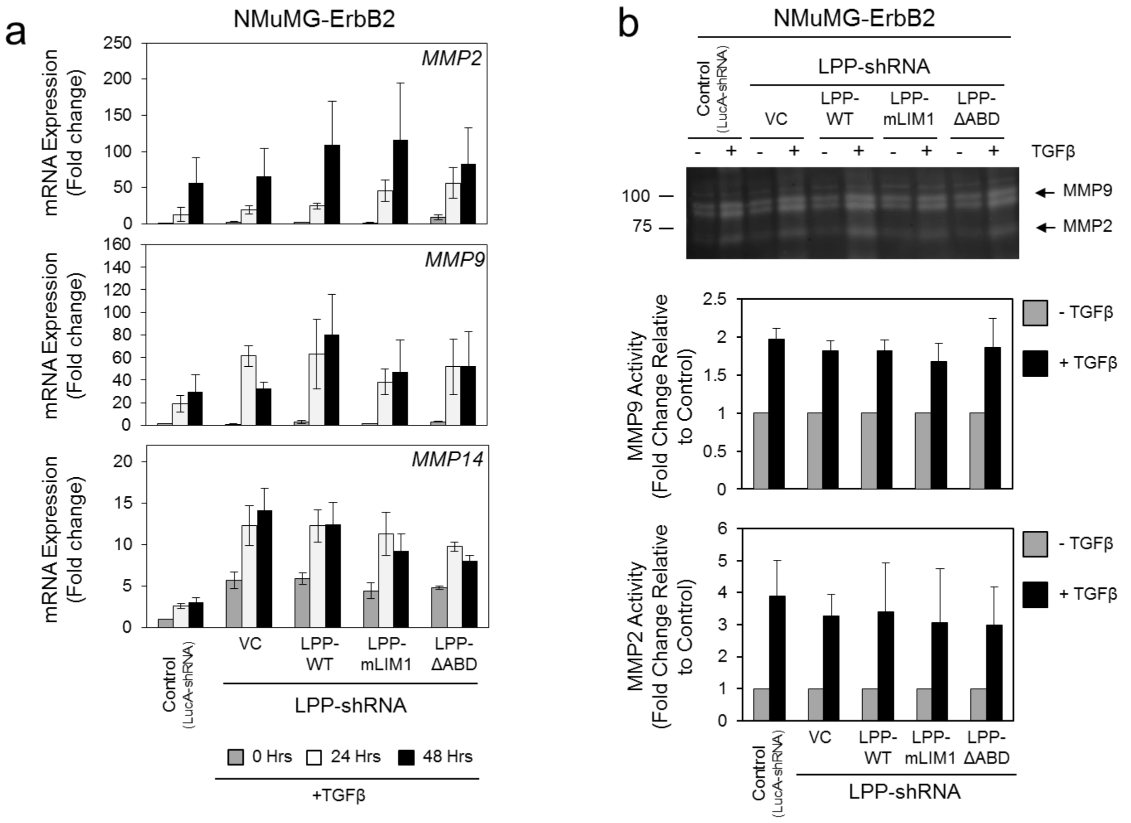

Supplementary Figure 7. MMP activity is unaffected by reduced LPP levels or the expression of LPP mutants. Total RNA was collected from the indicated cell lines that were treated with doxycycline, in the presence or absence of TGFβ for the indicated times. (a) Loss of LPP does not affect TGFβ-induced mRNA expression of *MMP2*, *MMP9* or *MMP14*. (b) MMP2 and MMP9 enzymatic activity was measured by gelatin zymography in conditioned media harvested from NMuMG-ErbB2 cells, treated with doxycycline and in the presence or absence of TGFβ (24 hrs). The TGFβ-induced activities of MMP2 and MMP9 were quantified and normalized to the respective untreated control (-TGFβ). The data represents the average of 3 independent experiments and the error bars represent s.e.m.

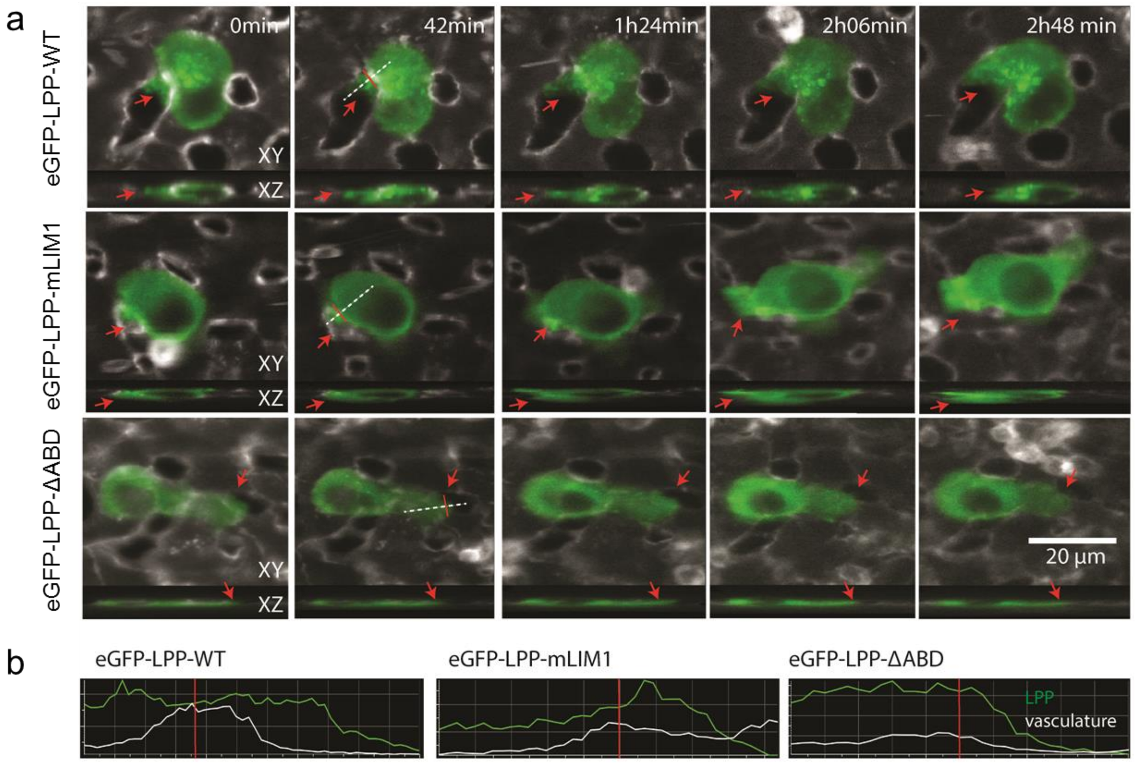

Supplementary Figure 8. Loss of LPP diminishes *ex ovo* tumor cell extravasation. (a) NMuMG-ErbB2 breast cancer cells with an LPP targeting shRNA and re-expressing eGFP-LPP-WT (*upper panel*), eGFP-LPP-mLIM1 (*middle panel*) and eGFP-LPP-ΔABD (*lower panel*) were intravenously injected into the CAM and monitored using high-resolution time-lapse intravital imaging. The luminal surface of the endothelium (grey signal) was labeled with A647-lectin. Red arrows point to the cancer cell/vascular wall breaching regions. (b) Line intensity scans of wild type and LPP-mutant cancer cell protrusions invading across the vascular wall. Line scans are shown for t = 42min (dashed white line). The red line indicates the approximate location of the vascular wall. See Supplementary Movie 1 for the full time-lapse sequence.

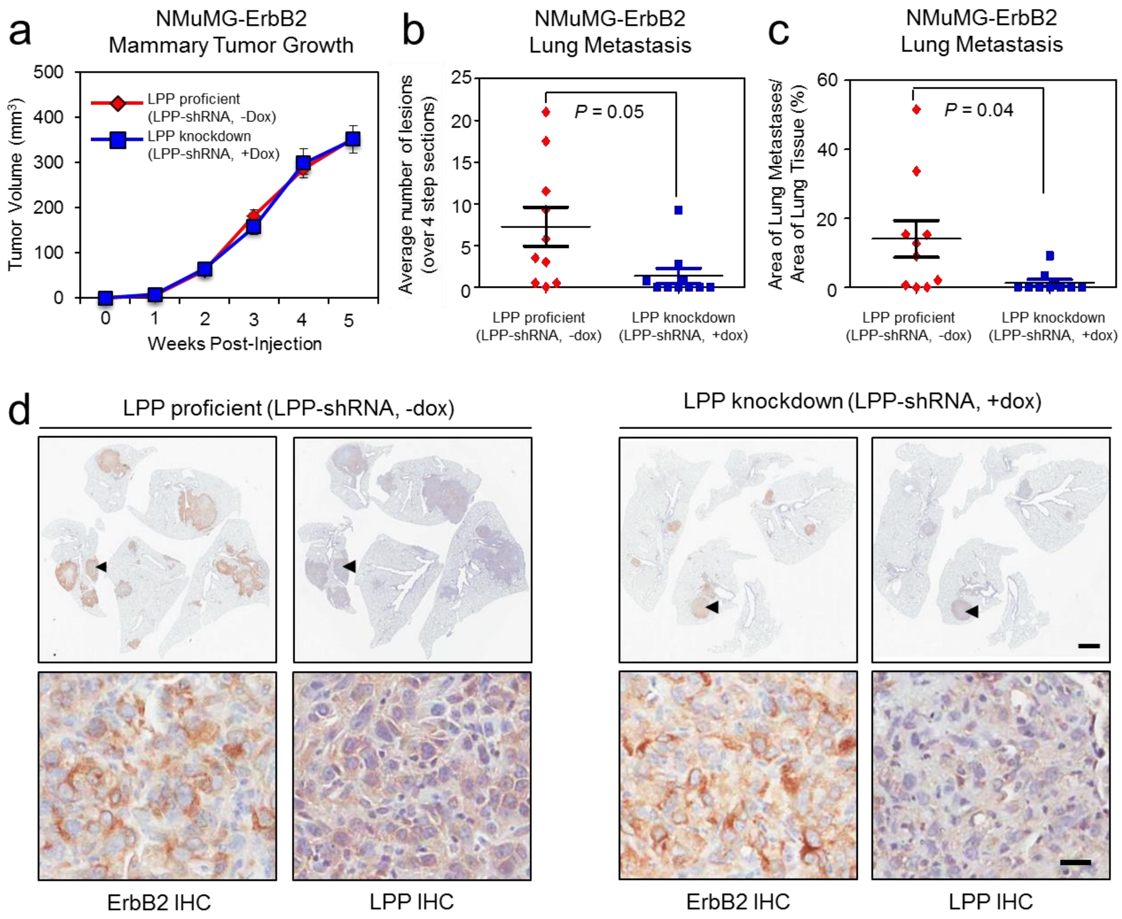

Supplementary Figure 9. Dox-inducible loss of LPP expression impairs lung metastasis formation. (a) NMuMG-ErbB2 cells harboring a doxycycline-inducible LPP shRNA were pre-treated in the presence (+dox: LPP knockdown) or absence (-dox: LPP proficient) of doxycycline prior to injection into the mammary fat pads of athymic mice. Cohorts of mice were administered oral dox or vehicle in the drinking water during the course of the experiment. Tumor growth was monitored by weekly caliper measurements. (b) NMuMG-ErbB2 cells expressing a dox-inducible shRNA against LPP were pre-treated in the presence (+dox: LPP knockdown) or absence (-dox: LPP proficient) of dox prior to injection into the tail veins of mice. Cohorts of mice were treated with (+dox) or without (-dox) oral doxycycline in the drinking water, which began 1 week prior to injection and continued throughout the duration of the experiment. Mice were sacrificed 9 weeks later and lung tissue collected at necropsy. The number of metastatic lesion was analyzed and the average number of lesions over 4 sections for each animal is shown (P = 0.05). (c) The area of metastatic lesions over total lung tissue area was quantified from dox-treated (LPP knockdown) (n = 10) and non-treated (LPP proficient) (n = 10) animals (P = 0.04). (d) Representative images of lungs harvested from vehicle (–dox) and treated (+dox) mice, stained by IHC against ErbB2 and LPP, are shown. The arrows indicate regions within the low magnification images that are shown in the insets. The scale bar within the low magnification images represents 2mm and applies to all images. The scale bar within the inset represents 20μm and applies to all inset images.

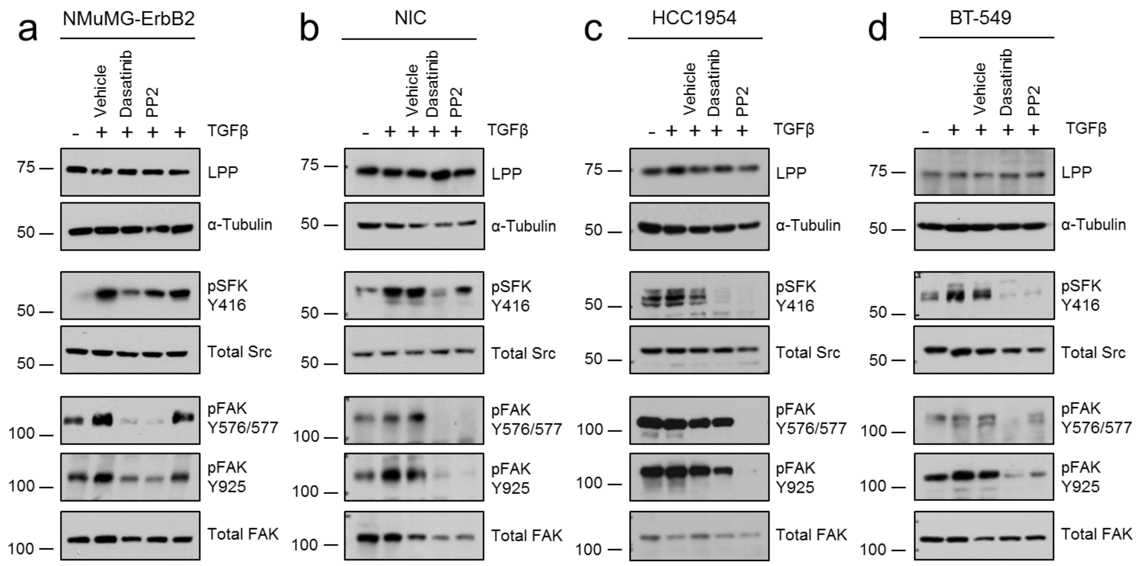

Supplementary Figure 10. Src Family Kinase (SFK) inhibitors impair TGFβ-induced Src-FAK pathway activation in breast cancer cell lines. NMuMG-ErbB2 (a), NIC (b), HCC1954 (c) and BT549 (d) breast cancer cell lines were stimulated with or without TGFβ and incubated in the presence of SFK inhibitors (Dasatinib, PP2) or a vehicle control (DMSO). Total cell lysates were immunoblotted with LPP, α-Tubulin and the indicated antibodies to assess inhibition of Src-FAK signaling pathways in response to pharmacological inhibitors. The same lysates were used for immunoprecipitation of LPP (Fig. 7a).

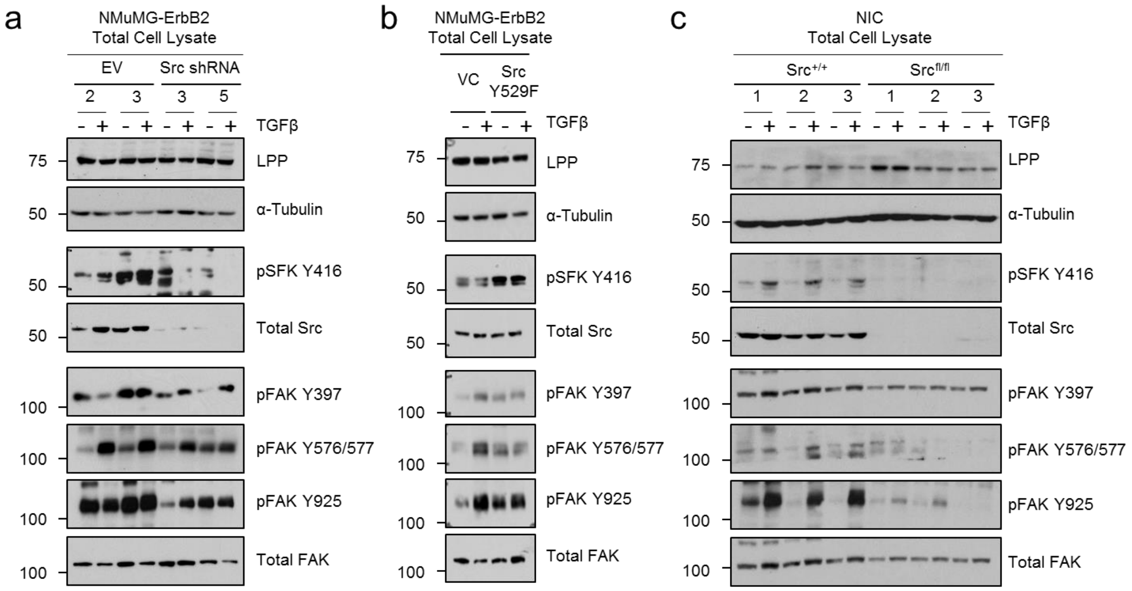

Supplementary Figure 11. Characterization of Src-FAK signaling in multiple breast cancer cells with diminished or elevated Src expression. Total cell lysates from breast cancer cells stimulated with or without TGFβ were immunoblotted with LPP, α-Tubulin and the indicated antibodies to assess inhibition of Src-FAK signaling pathways in (a) NMuMG-ErbB2 breast cancer cells infected with Src-shRNAs or Empty Vector controls (EV), (b) NMuMG-ErbB2 breast cancer cells transfected with a constitutively activated Src construct or (c) 3 independent NIC $Src^{+/+}$ and $Src^{fl/fl}$ breast cancer cell lines.

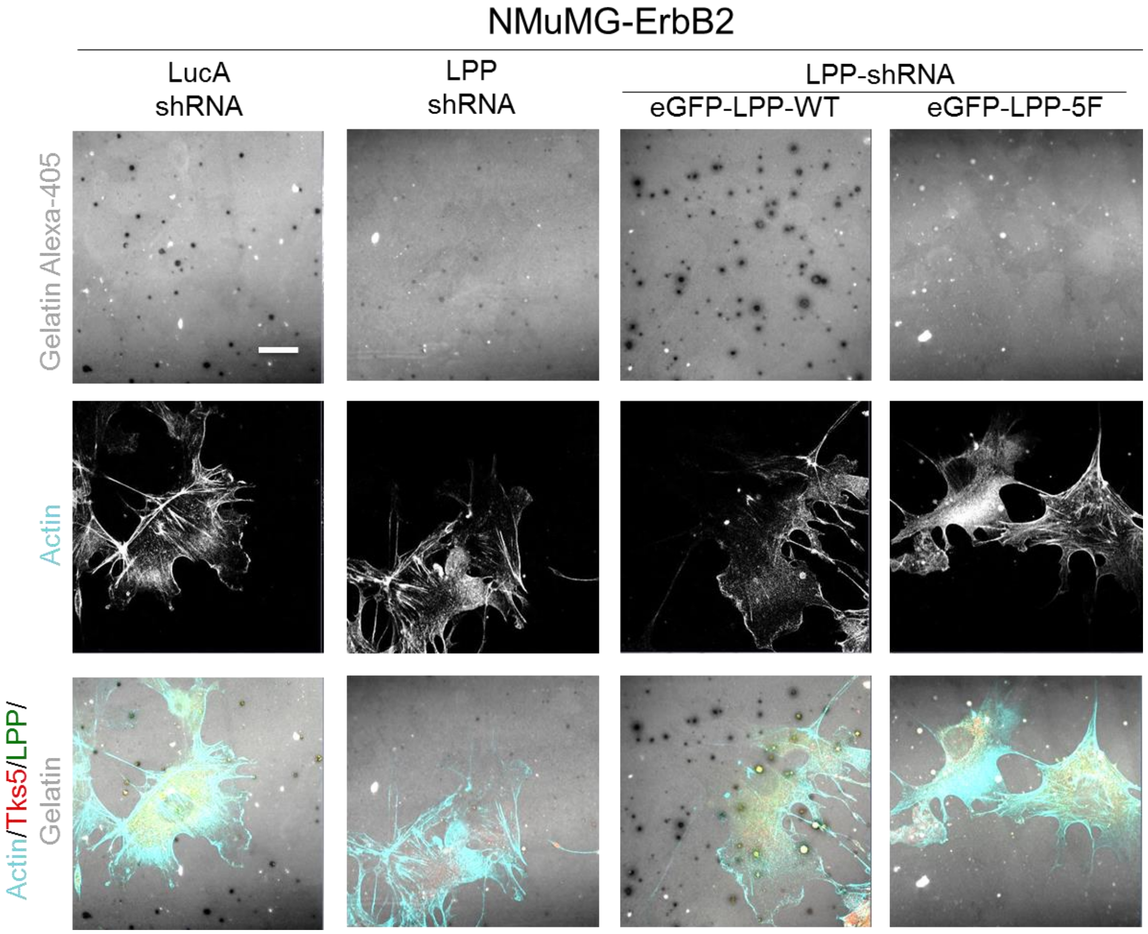

Supplementary Figure 12. Phosphorylation of LPP on key tyrosine residues is required for breast cancer cell mediated gelatin degradation. (a) NMuMG-ErbB2 cells expressing LucA-shRNA (control) and LPP-shRNA (LPP knockdown), along with with rescue constructs (eGFP-LPP-WT or eGFP-LPP-5F), were treated with TGFβ (24 hours) and plated onto Alexa 405 labeled gelatin. After 24 hours, cells were fixed and stained for LPP (in control and knockdown cells), Tks5 and Actin (phalloidin) to visualize their localization on the gelatin substrate. The scale bar = 20μm, and applies to all images.

Figure 3a

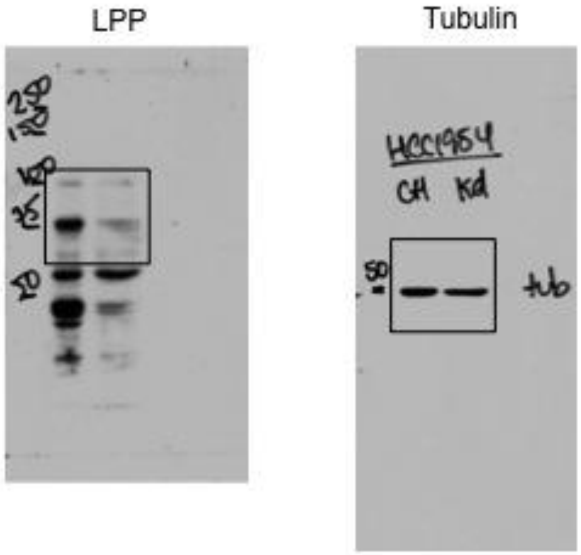

Figure 6a

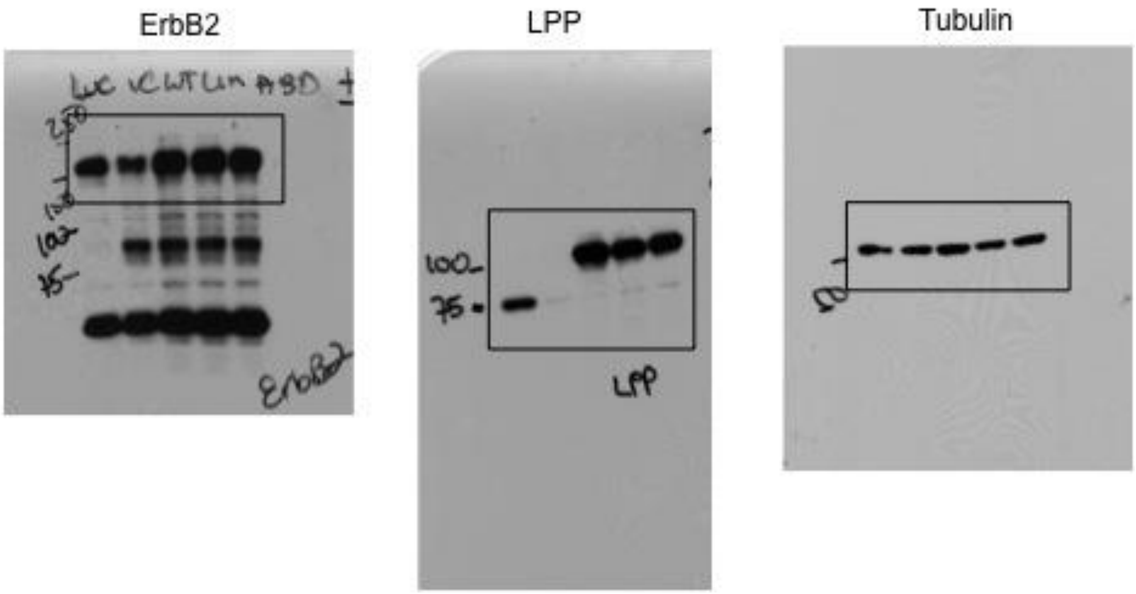

Figure 7a

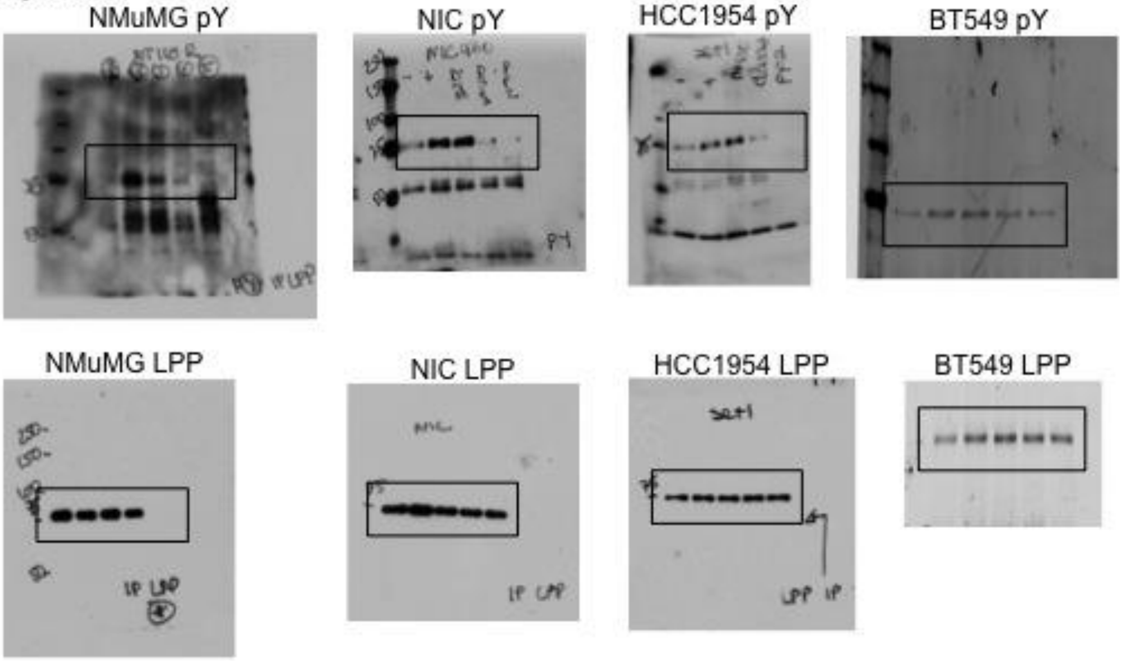

Figure 7b

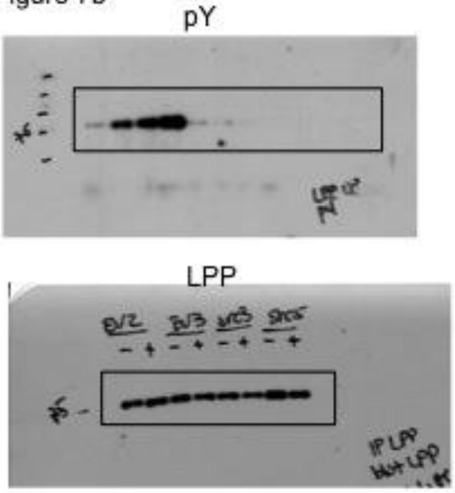

Figure 7c

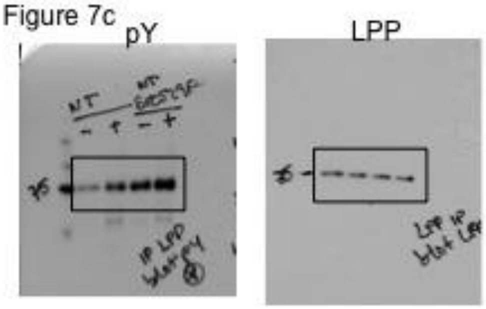

Figure 7d

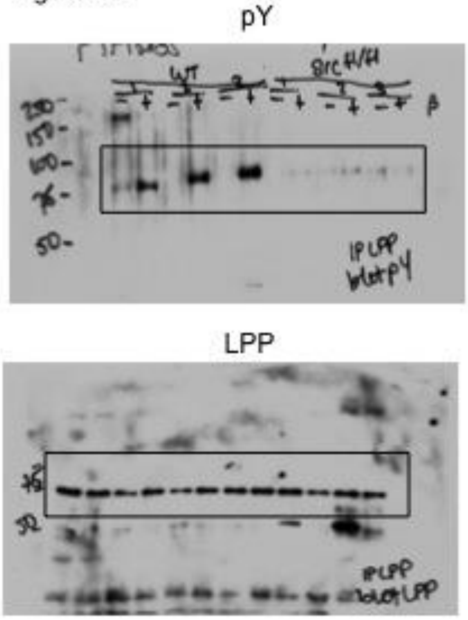

Figure 8b

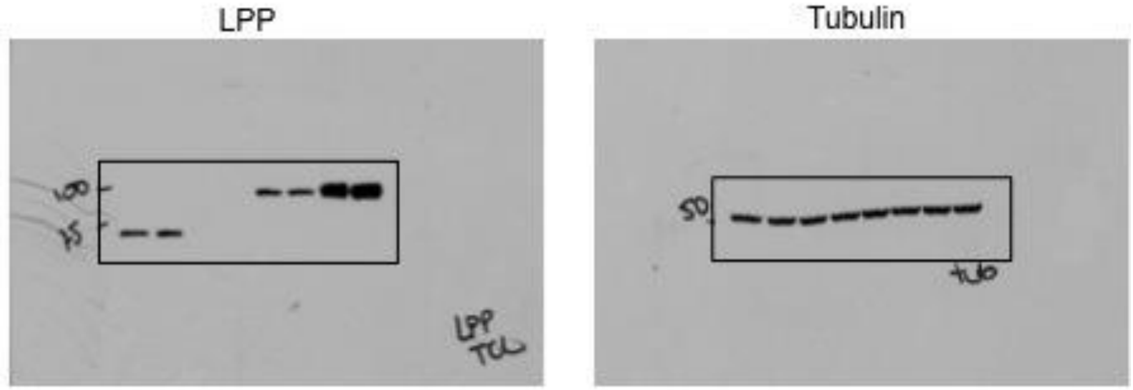

Figure 9b

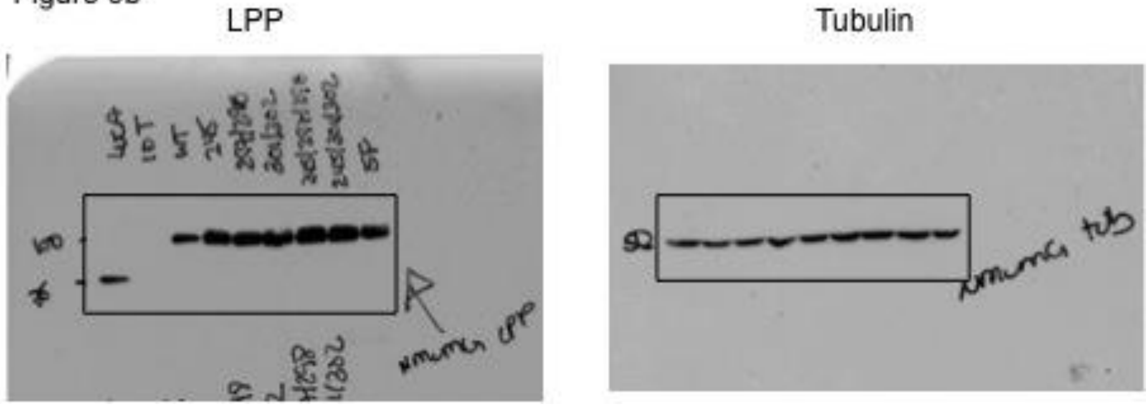

Supplementary Figure 13. Uncropped scans of all immunoblots presented in the main article. The cropped sections used in each figure presented in the main article are indicated by black rectangles.
